# Supplementary material for: HR-MAS NMR Metabolomics Profile of Vero Cells under the Influence of Virus Infection and nsP2 Inhibitor: A Chikungunya Case Study
Source: Int J Mol Sci. 2024 Jan 24;25(3):1414. doi: 10.3390/ijms25031414 (PMC10855909; doi:10.3390/ijms25031414)
Supplement: Supplementary file 1 [file ijms-25-01414-s001.zip › ijms-2803141-supplementary.pdf]

Supplementary information

HR-MAS NMR Metabolomics profile of Vero cells under the influence of virus infection and nsP2 inhibitor: A Chikungunya Case Study

Rafaela dos S. Peinado[a†], Lucas G. Martins[b†], Carolina C. Pacca[c,d], Marielena V. Saivish[c], Kelly C. Borsatto[a], Maurício L. Nogueira[c], Ljubica Tasic[b], Raghuvir K. Arni[a*], Raphael J. Eberle[e,f*], Mônika A. Coronado[e*].

[a]Multiuser Center for Biomolecular Innovation, Department of Physics - Institute of Biosciences, Languages and Exact Sciences (Ibilce - UNESP), Sao Jose do Rio Preto – Sao Paulo, Brazil.

[b]Department of Organic Chemistry, Institute of Chemistry, University of Campinas (UNICAMP), Campinas, Brazil.

[c]Virology Research Laboratory, Medical School of Sao Jose do Rio Preto (FAMERP) – Sao Paulo, Brazil.

[d]FACERES Medical School, Sao Jose do Rio Preto – Sao Paulo, Brazil.

[e]Institute of Biological Information Processing (IBI-7: Structural Biochemistry), Forschungszentrum Jülich, Jülich, Germany.

[f]Institut für Physikalische Biologie, Heinrich-Heine-Universität Düsseldorf, Düsseldorf, Germany.

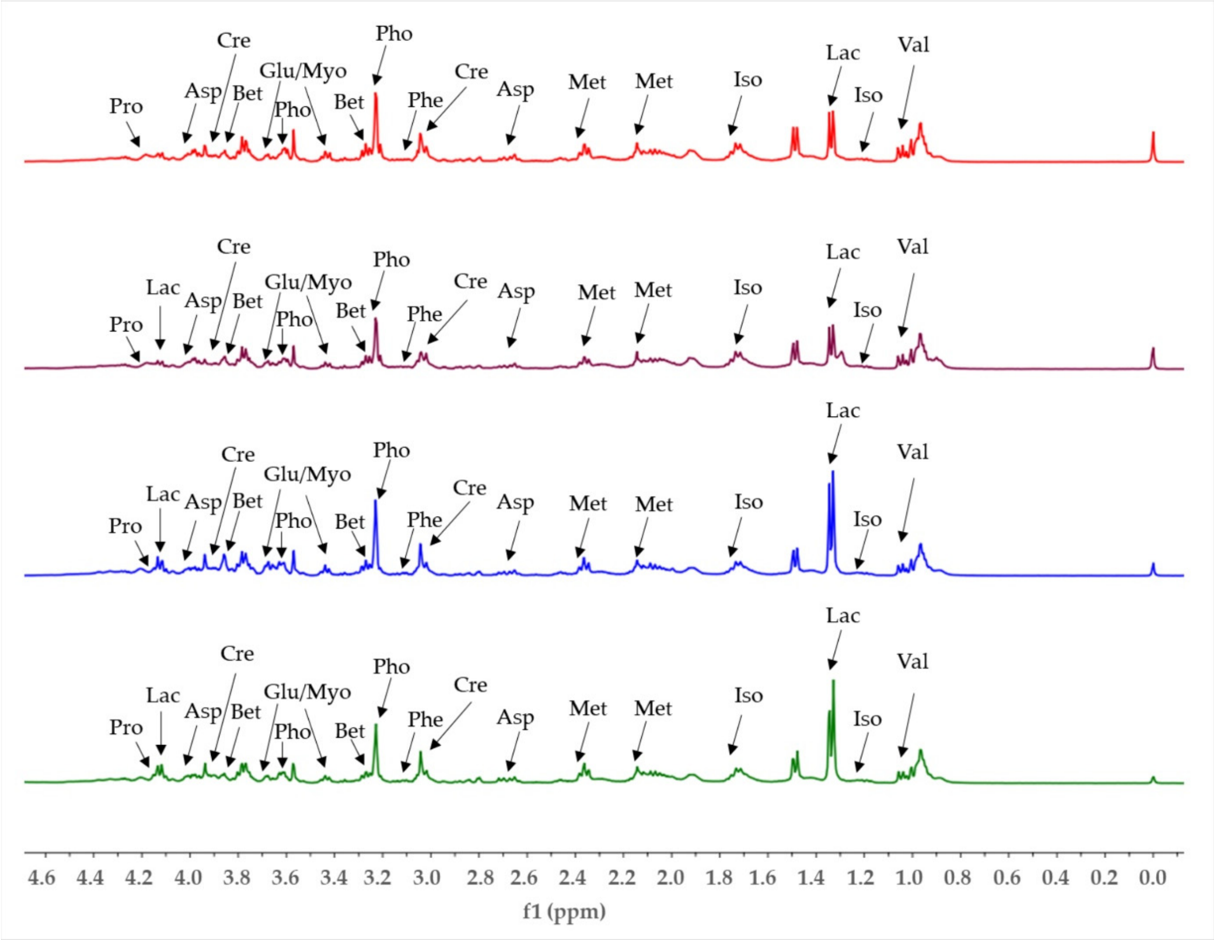

Figure S1. HR-MAS ^{1}H-NMR T_2-edited (CPMG) spectra (400 MHz) of all four conditions demonstrating the most differential metabolites among them. Vero E6 control sample spectrum is represented in green (CC), cells treated with WDL (CW) are in blue, cells infected and treated with the molecule (CWV) are represented in purple, and CHIKV-infected cells are represented in red (CV). Identified metabolites are Val (Valine); Lac (Lactate); Iso (Isoleucine); Pro (Proline); Met (Methionine); Asp (Aspartate); Cre (Creatine); Phe (Phenylalanine); Pho (Phosphocholine); Glu (Glucose); Bet (Betaine); Myo (Myo-inositol).

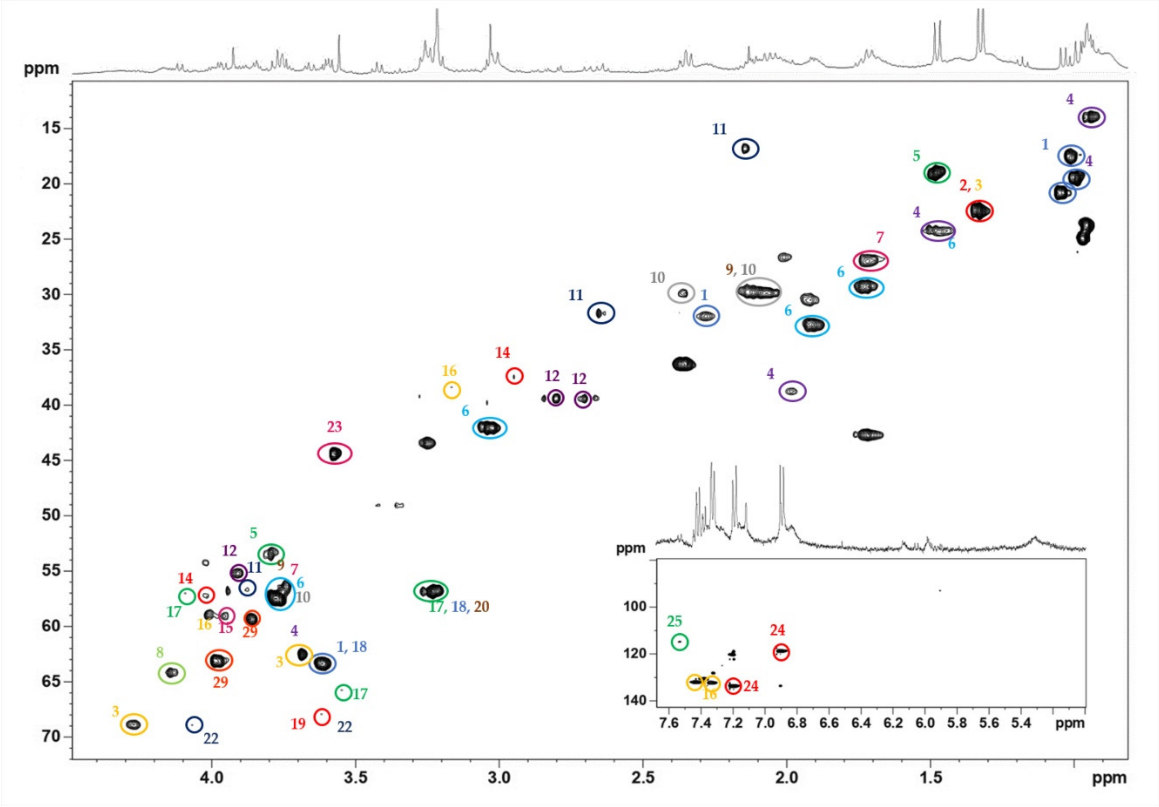

Figure S2. HR-MAS 2D-HSQC spectrum of Vero E6 cells samples acquired on a Bruker Avance III 400 MHz. The image of the spectrum comprises the region between 0 and 4.50 ppm, amplified 20 times, and the small image on the right corner shows the 5.20 to 7.60 ppm region for 1H chemical shifts and from 10 to 70 ppm for 13C. Identified metabolites are numbered according to Figure S1 and Table S1.

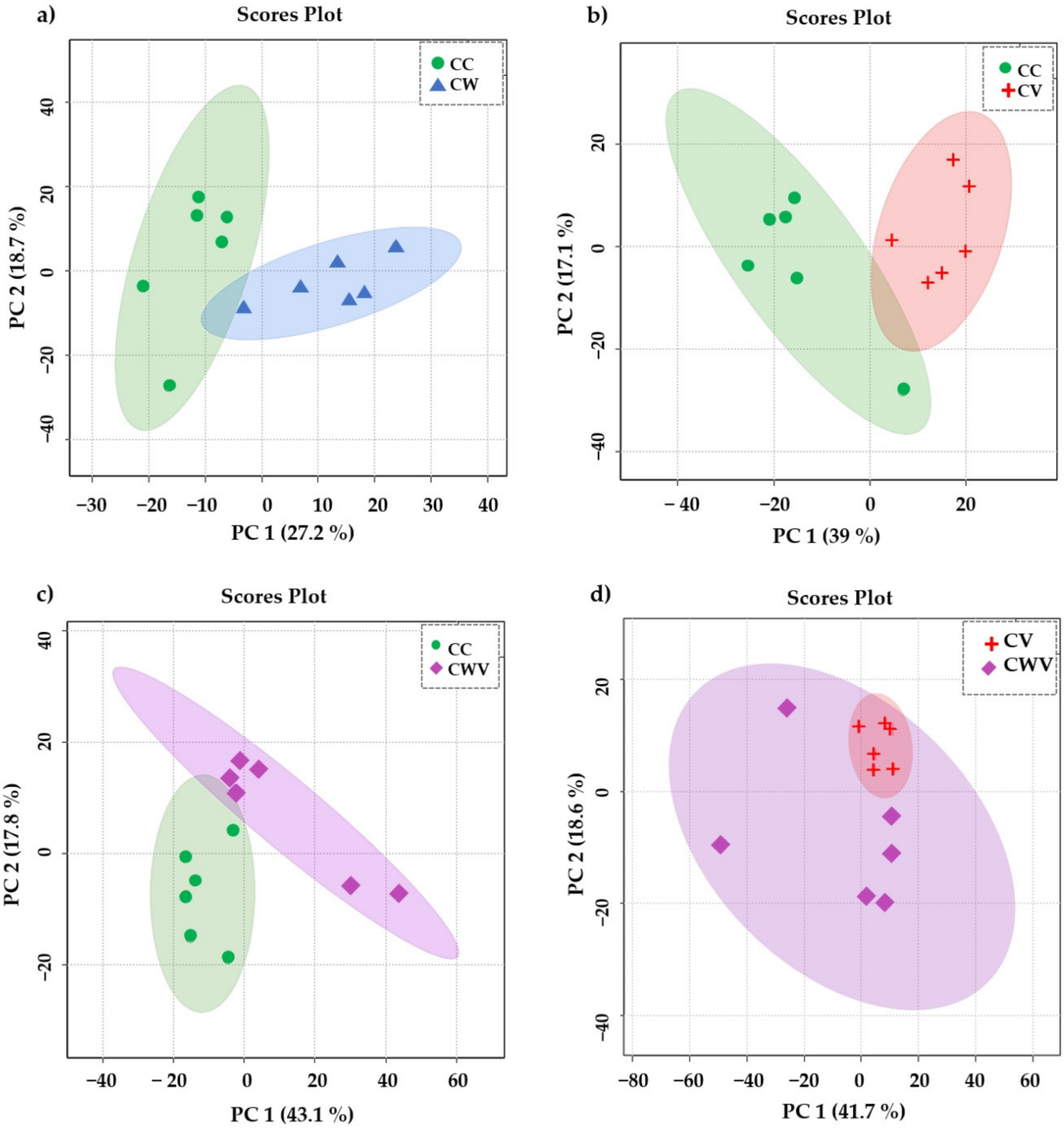

Figure S3. PCA scores plot in 2D of the HR-MAS 1H-NMR data showing the intrinsic groupings of the conditions compared to the healthy cell control (CC), shown in green, and no outliers. (**a**) The cells treated with WDL (CW) in blue, (**b**) the cells infected with CHIKV in red (CV), and (**c**) the CHIKV-infected cells treated with WDL in purple (CWV). The explained variance with the first two principal components is shown in the y and x axes.

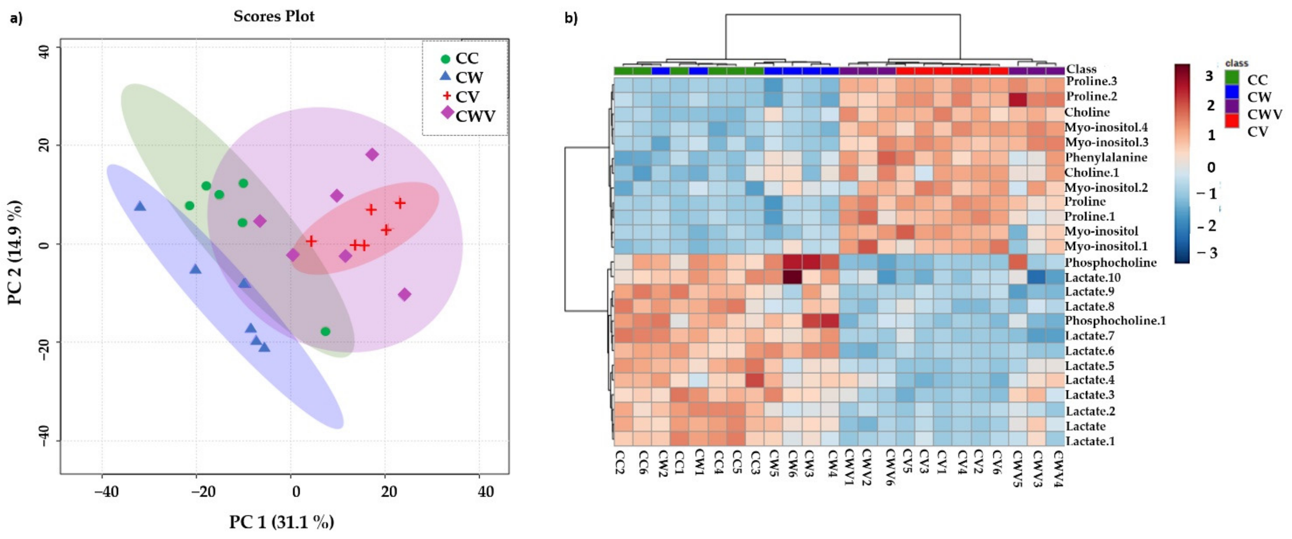

Figure S4. (**a**) PCA scores plot in 2D of the HR-MAS ^{1}H-NMR data showing the intrinsic groupings of all the conditions: the healthy cell control (CC), shown in green, the cells treated with WDL (CW) in blue, the cells infected with CHIKV in red (CV), and (**c**) the CHIKV-infected cells treated with WDL in purple (CWV). The explained variance with the first two principal components is shown in the y and x axes. (**b**) Hierarchical clustering through heat maps analysis using Euclidean distance. The lines represent the discriminant variables from the PLS-DA analysis for all conditions CC × CV × CW × CWV. The columns represent the samples, and the colour bars on the right represent the relative concentrations of the discriminant metabolites (red: higher relative concentration; blue: lower relative concentration).

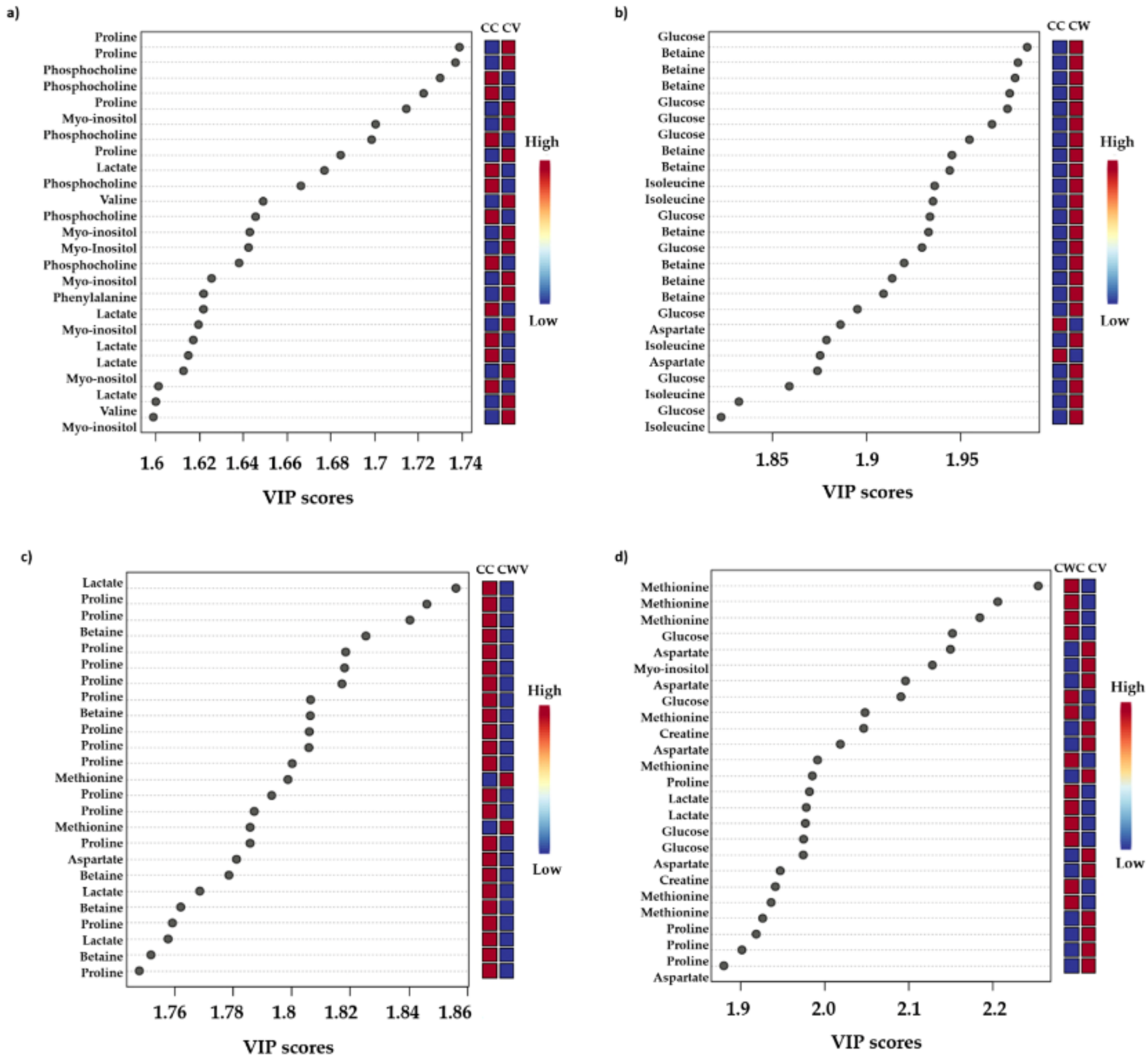

Figure S5. VIP plots of the most discriminant variables from the PLS-DA models, with scores > 1.5. (**a**) CC × CV; (**b**) CC × CW; (**c**) CC × CWV; (**d**) CV × CWV .

Table S1. Cell toxicity of WDL in Vero E6 cell lineage.

Concentration	Cell Viability (%)*
**400 µM**	45.5 ± 17.0
**300 µM**	59.0 ± 13.0
**200 µM**	68.0 ± 8.0
**100 µM**	74.7 ± 14.0
**50 µM**	93.6 ± 10.1

* Values with 95% confidence intervals (CI).

Table S2. Chemical shifts, spectral peaks multiplicities and coupling constants of some of the most important metabolites.

Metabolite	Nº	^{1}H Chemical shifts (ppm), spectral peaks multiplicities and coupling constant	^{13}C Chemical shifts (ppm), spectral peaks multiplicities and coupling constant
**Valine**	1	0.97 d (*J* = 8.0 Hz); 1.05 d (*J* = 7.0 Hz); 2.28 m; 3.61 d (*J* = 4.0 Hz)	17.1, 19.3, 20.5, 31.6, 63.2
**Lactate**	2	1.33 d (*J* = 7.0 Hz); 4.12 q (*J* = 7.0 Hz)	22.4
**Threonine**	3	1.33 d (J = 6.7 Hz); 3.60 (J = 6.7 Hz); 4.26 m	22.6, 63.2, 68.8
**Isoleucine**	4	0.93 t (*J* = 8.0 Hz); 0.99 d (*J* = 9.0 Hz); 1.24 m; 1.46 m; 1.97 m; 3.67 d (*J* = 4.0 Hz)	14.1, 17.1, 24.1, 38.6, 62.5
**Alanine**	5	1.48 d (*J* = 7.3 Hz); 3.79 q (*J* = 7.3 Hz)	18.8, 53.1
**Lysine**	6	1.43 m; 1.73 m; 1.91 m; 3.03 t; 3.75 t (*J* = 6.1 Hz)	24.1, 29.2, 32.6, 42.1, 57.4
**Arginine**	7	1.68 m (*J* = 7.0 Hz); 1.90 m (*J* = 7.3 Hz); 3.25 t (*J* = 6.9 Hz); 3.75 t (*J* = 6.1 Hz)	26.4, 30.4, 43.3, 57.2
**Proline**	8	2.10 m; 2.36 m 3.58m; 4.14 dd (*J* = 8.6, 6.4 Hz)	31.1, 64.2
**Glutamate**	9	2.12 m; 2.37 m; 3.77 dd (*J* = 7.2, 4.7 Hz)	30.2, 31.6, 56.5
**Glutamine**	10	2.14 m; 2.45 m; 3.77 t (*J* = 6.2 Hz)	29.4, 33.8, 57.2
**Methionine**	11	2.14 m; 2.17 s; 2.65 t (*J* = 7.6 Hz); 3.85 dd (*J*=7.1, 5.4 Hz)	16.6, 32.3, 31.5, 56.6
**Aspartate**	12	2.68 dd (*J* = 17.5, 8.9 Hz); 2.82 dd (*J* = 17.5, 3.7 Hz); 3.92 dd (*J* = 8.8, 3.8 Hz)	39.3, 55.1
**Hypotaurine**	13	2.55 t (*J* = 6.8 Hz); 3.23 t (*J* = 6.8 Hz)	-
**Asparagine**	14	2.88 m; 2.94 m; 3.99 dd (*J* = 7.7, 4.3 Hz)	37.4, 37.6, 54.1
**Creatine**	15	3.04 s; 3.93 s	59.1
**Phenylalanine**	16	3.11 dd (*J* = 14.6, 8.0 Hz); 3.28 dd (*J* = 14.6, 5.5 Hz); 4.00 dd (*J*	39.1, 58.9, 132.2, 131.7, 131.4

		= 7.9, 5.3 Hz); 7.33 m; 7.39 m; 7.42 m	
**Choline**	17	3.21 s; 3.51 dd (*J* = 5.8, 4.2 Hz); 4.06 dd	56.5, 70.2, 58.1
**Phosphocholine**	18	3.23 s; 3.62 m; 4.22 m	56.7, 64.1
**Glucose**	19	3.31 m; 3.44 m; 3.54 m	74.1
**Betaine**	20	3.26 s; 3.89 s	56.8
**Trimethylamine-N-oxide**	21	3.27 s	-
**Myo-inositol**	22	3.27 t (*J* = 11.8 Hz); 3.52 dd (*J* = 9.9, 2.9); 3.61 t (*J* = 9.7 Hz); 4.05 t (*J* = 2.9 Hz)	77.2, 73.8, 75, 74.9
**Glycine**	23	3.57 s	44.3
**Tyrosine**	24	6.89 m; 7.19 m	118.8, 133.6
**Tryptophan**	25	7.53 m; 7.72 m	115.1, 121.3
**UDP-N-acetylglucosamine**<br>**UDP-glucose**<br>**UDP-glucuronate**	26	5.97 d; 7.95 d	144.2
**Fumarate**	27	6.53 s	-
**ATP**	28	6.15 d; 8.26 s; 8.56 s	-
**Serine**	29	3.86 dd; 3.97 m	59.2, 63.3

Table S3. Discriminant metabolites from the univariate analysis of CC × CV analysis (*T-test*, Fisher’s LSD post-hoc test, $p < 0.05$).

**Metabolite**	**t.stat**	**p.value**	**-log10(p)**	**FDR**
**Proline**	-19.18	3.21E-09	8.49	1.88E-06
**Proline**	-18.47	4.64E-09	8.33	1.88E-06
**Phosphocholine**	16.29	1.57E-08	7.80	4.25E-06
**Phosphocholine**	14.61	4.48E-08	7.34	9.07E-06
**Proline**	-13.30	1.09E-07	6.95	1.77E-05
**Myo-inositol**	-11.62	3.93E-07	6.40	5.25E-05

**Phosphocholine**	11.44	4.54E-07	6.34	5.25E-05
**Proline**	-10.28	1.22E-06	5.91	0.00012
**Lactate**	9.79	1.91E-06	5.71	0.00017
**Phosphocholine**	9.18	3.43E-06	5.46	0.00027
**Valine**	-8.39	7.73E-06	5.11	0.00056
**Phosphocholine**	8.25	8.95E-06	5.04	0.00059
**Myo-inositol**	-8.15	1.00E-05	5.00	0.00059
**Myo-inositol**	8.12	1.02E-05	4.99	0.00059
**Phosphocholine**	7.96	1.21E-05	4.91	0.00065
**Myo-inositol**	-7.55	1.94E-05	4.71	0.00098
**Phenylalanine**	-7.43	2.21E-05	4.65	0.00099
**Lactate**	7.43	2.22E-05	4.65	0.00099
**Myo-inositol**	-7.36	2.39E-05	4.62	0.00102
**Lactate**	7.30	2.59E-05	4.58	0.00105
**Lactate**	7.23	2.80E-05	4.55	0.00108
**Myo-inositol**	-7.17	3.01E-05	4.52	0.00110
**Lactate**	6.87	4.30E-05	4.36	0.00145
**Valine**	-6.84	4.46E-05	4.35	0.00145
**Myo-inositol**	-6.82	4.61E-05	4.33	0.00145

Table S4. Discriminant metabolites from the univariate analysis of CC × CW analysis (*T-test*, Fisher's LSD post-hoc test, $p < 0.05$).

**Metabolite**	**t.stat**	**p.value**	**-log10(p)**	**FDR**
**Glucose**	-13.91	7.16E-8	7.14	2.95E-5
**Betaine**	-13.26	1.13E-7	6.94	2.95E-5
**Betaine**	-13.07	1.29E-7	6.88	2.95E-5
**Betaine**	-12.74	1.65E-7	6.78	2.95E-5
**Glucose**	-12.61	1.82E-7	6.73	2.95E-5
**Glucose**	-11.80	3.41E-7	6.46	4.60E-5
**Glucose**	-10.84	7.49E-7	6.12	8.67E-5
**Betaine**	-10.24	1.27E-6	5.89	1.22E-4
**Betaine**	-10.16	1.36E-6	5.86	1.22E-4
**Isoleucine**	-9.72	2.05E-6	5.68	1.50E-4

**Isoleucine**	-9.67	2.15E-6	5.66	1.50E-4
**Glucose**	-9.59	2.32E-6	5.63	1.50E-4
**Betaine**	-9.55	2.40E-6	5.61	1.50E-4
**Glucose**	-9.38	2.83E-6	5.54	1.64E-4
**Betaine**	-8.95	4.32E-6	5.36	2.33E-4
**Betaine**	-8.70	5.60E-6	5.25	2.83E-4
**Betaine**	-8.52	6.71E-6	5.17	3.20E-4
**Glucose**	-8.04	1.11E-5	4.95	5.02E-4
**Aspartate**	7.77	1.50E-5	4.82	6.43E-4
**Isoleucine**	-7.56	1.91E-5	4.71	7.73E-4
**Aspartate**	7.47	2.11E-5	4.67	8.14E-4
**Glucose**	-7.43	2.21E-5	4.65	8.14E-4
**Isoleucine**	-7.07	3.39E-5	4.46	0.0011
**Glucose**	-6.52	6.71E-5	4.17	0.0022
**Isoleucine**	-6.34	8.36E-5	4.07	0.0026

Table S5. Discriminant metabolites from the univariate analysis of CC × CWV analysis (*T-test*, Fisher's LSD post-hoc test, $p < 0.05$).

**Metabolite**	**t.stat**	**p.value**	**-log10(p)**	**FDR**
**Lactate**	10.00	1.58E-6	5.79	9.43E-4
**Proline**	9.45	2.64E-6	5.57	9.43E-4
**Proline**	9.17	3.48E-6	5.45	9.43E-4
**Betaine**	8.53	6.66E-6	5.17	0.0010
**Proline**	8.27	8.73E-6	5.05	0.0010
**Proline**	8.26	8.86E-6	5.05	0.0010
**Proline**	8.23	9.16E-6	5.03	0.0010
**Proline**	7.87	1.35E-5	4.86	0.0010
**Betaine**	7.87	1.35E-5	4.86	0.0010
**Proline**	7.86	1.37E-5	4.86	0.0010
**Proline**	7.85	1.37E-5	4.86	0.0010
**Proline**	7.68	1.67E-5	4.77	0.0010
**Methionine**	-7.64	1.75E-5	4.75	0.0010
**Proline**	7.48	2.10E-5	4.67	0.0012
**Proline**	7.32	2.52E-5	4.59	0.0012
**Methionine**	-7.28	2.63E-5	4.57	0.0012
**Proline**	7.28	2.63E-5	4.57	0.0012
**Aspartate**	7.17	3.02E-5	4.51	0.0013
**Betaine**	7.10	3.26E-5	4.48	0.0013
**Lactate**	6.87	4.31E-5	4.36	0.0017
**Betaine**	6.73	5.13E-5	4.28	0.0019
**Proline**	6.67	5.53E-5	4.25	0.0020
**Lactate**	6.64	5.74E-5	4.24	0.0020
**Betaine**	6.52	6.65E-5	4.17	0.0022
**Proline**	6.45	7.34E-5	4.13	0.0023

Table S6. Discriminant metabolites from the univariate analysis of CV × CWV analysis (*T-test*, Fisher's LSD post-hoc test, $p < 0.05$).

Metabolite	t.stat	p.value	-log10(p)	FDR
**Methionine**	9.57	2.36E-6	5.62	0.001
**Methionine**	7.95	1.23E-5	4.90	0.004
**Methionine**	7.44	2.20E-5	4.65	0.005
**Glucose**	6.79	4.76E-5	4.32	0.008
**Aspartate**	-6.75	5.01E-5	4.29	0.008
**Myo-inositol**	-6.40	7.79E-5	4.10	0.010
**Aspartate**	-5.95	1.40E-4	3.85	0.010
**Glucose**	5.88	1.54E-4	3.81	0.015
**Methionine**	5.39	3.01E-4	3.52	0.024
**Creatine**	-5.38	3.08E-4	3.51	0.024
**Aspartate**	-5.11	4.53E-4	3.34	0.033
**Methionine**	4.87	6.43E-4	3.19	0.035
**Proline**	-4.82	6.93E-4	3.15	0.035
**Lactate**	4.80	7.22E-4	3.14	0.035
**Lactate**	4.77	7.56E-4	3.12	0.035
**Glucose**	4.76	7.65E-4	3.11	0.035
**Glucose**	4.74	7.85E-4	3.10	0.035
**Aspartate**	-4.74	7.89E-4	3.10	0.035
**Creatine**	-4.53	0.001	2.96	0.045
**Methionine**	4.49	0.001	2.94	0.046
**Methionine**	4.46	0.001	2.91	0.046
**Proline**	4.39	0.001	2.87	0.049
